# Cdc14 phosphatases use an intramolecular pseudosubstrate motif to stimulate and regulate catalysis

**DOI:** 10.1016/j.jbc.2024.107644

**Published:** 2024-08-08

**Authors:** Kedric L. Milholland, Benjamin T. Waddey, Kevin G. Velázquez-Marrero, Michelle V. Lihon, Emily L. Danzeisen, Noelle H. Naughton, Timothy J. Adams, Jack L. Schwartz, Xing Liu, Mark C. Hall

**Affiliations:** 1 Department of Biochemistry, Purdue University, West Lafayette, Indiana, USA; 2 Center for Plant Biology, Purdue University, West Lafayette, Indiana, USA; 3 Institute for Cancer Research, Purdue University, West Lafayette, Indiana, USA; 4 Institute for Drug Discovery, Purdue University, West Lafayette, Indiana, USA; 5 Institute of Inflammation, Immunology, and Infectious Disease, Purdue University, West Lafayette, Indiana, USA

**Keywords:** Cdc14, phosphatase, pseudosubstrate, phosphorylation, enzyme catalysis, enzyme mechanism, enzyme kinetics, enzyme regulation, structural model

## Abstract

Cdc14 phosphatases are related structurally and mechanistically to protein tyrosine phosphatases (PTPs) but evolved a unique specificity for phosphoSer-Pro-X-Lys/Arg sites primarily deposited by cyclin-dependent kinases. This specialization is widely conserved in eukaryotes. The evolutionary reconfiguration of the Cdc14 active site to selectively accommodate phosphoSer-Pro likely required modification to the canonical PTP catalytic cycle. While studying *Saccharomyces cerevisiae* Cdc14, we discovered a short sequence in the disordered C terminus, distal to the catalytic domain, which mimics an optimal substrate. Kinetic analyses demonstrated this pseudosubstrate binds the active site and strongly stimulates rate-limiting phosphoenzyme hydrolysis, and we named it “substrate-like catalytic enhancer” (SLiCE). The SLiCE motif is found in all Dikarya fungal Cdc14 orthologs and contains an invariant glutamine, which we propose is positioned *via* substrate-like contacts to assist orientation of the hydrolytic water, similar to a conserved active site glutamine in other PTPs that Cdc14 lacks. AlphaFold2 predictions revealed vertebrate Cdc14 orthologs contain a conserved C-terminal alpha helix bound to the active site. Although apparently unrelated to the fungal sequence, this motif also makes substrate-like contacts and has an invariant glutamine in the catalytic pocket. Altering these residues in human Cdc14A and Cdc14B demonstrated that it functions by the same mechanism as the fungal motif. However, the fungal and vertebrate SLiCE motifs were not functionally interchangeable, illuminating potential active site differences during catalysis. Finally, we show that the fungal SLiCE motif is a target for phosphoregulation of Cdc14 activity. Our study uncovered evolution of an unusual stimulatory pseudosubstrate motif in Cdc14 phosphatases.

*CDC14* was originally identified in *Saccharomyces cerevisiae* as an essential gene required for the final stage of nuclear division (1). The Cdc14 protein was subsequently characterized as a protein phosphatase that fulfilled its essential function by terminating mitotic cyclin-dependent kinase (Cdk) activity to trigger mitotic exit (2, 3). Cdc14 homologs have now been identified and studied in a wide range of eukaryotes and implicated in diverse biological roles (4, 5, 6). For example, the Cdc14 homolog in *Schizosaccharomyces pombe*, Clp1/Flp1, is not required for mitotic exit, but rather regulates entry into mitosis and helps coordinate cytokinesis and septation with nuclear division (7, 8). Vertebrates evolved two Cdc14 paralogs and early siRNA-induced knockdown, protein depletion, and overexpression studies suggested various roles regulating cell division events, including chromosome segregation, centrosome and spindle function, the G2 DNA damage checkpoint, and cytokinesis (9, 10, 11, 12, 13). However, subsequent studies using gene KOs in zebrafish and avian, mouse, and human cell lines revealed that Cdc14 paralogs have little effect on cell division, but instead function in other processes like DNA repair, cell migration, and actin and cilia regulation (14, 15, 16, 17, 18, 19, 20). A recent report on mice lacking both CDC14A and B confirmed the absence of major cell cycle functions but uncovered a critical role in neural stem cell differentiation during early development (21). It is noteworthy that the double KO mice that survived the perinatal period had a normal lifespan in the complete absence of Cdc14 activity.

In pathogenic fungi, in addition to contributions to cell cycle processes like cytokinesis and septation, Cdc14 orthologs have been identified as critical virulence factors. This includes in the phytopathogens *Fusarium graminearum*, *Magnaporthe oryzae*, and *Aspergillus flavus* (22, 23, 24) and the entomopathogen *Beauveria bassiana* (25). Early work in the human pathogenic yeast, *Candida albicans*, showed that Cdc14 was important for normal hyphal development (26), a process critical for virulence (27). Consistent with this, we recently found that *C. albicans* Cdc14 is required for virulence in animal models of invasive candidiasis (28). These studies suggest a conserved role for Cdc14 in one or more biological processes specifically important for host infection. While the mechanism and relevant substrates remain unclear, the broad virulence requirement suggests that Cdc14 could be an effective target for the development of antifungal agents. The fact that angiosperm plants lack *CDC14* (29, 30, 31) makes it a particularly attractive target for crop pesticide development. However, since Cdc14 absence appears to have minimal impact on growth and development in metazoans (21, 32, 33), it could also have utility as an antifungal drug target to treat human infections. Nonetheless, a complete understanding of Cdc14 biochemistry and identification of exploitable structural and mechanistic differences between fungal and vertebrate Cdc14 enzymes would be beneficial for drug development.

Cdc14’s unique substrate specificity supports its potential as an antifungal target as well. Cdc14 is related to the dual-specificity phosphatase (DSP) subfamily of protein tyrosine phosphatases (PTPs), sharing the signature HCX_5_R catalytic motif and two-step reaction mechanism involving a phosphoenzyme intermediate (2, 34). However, it evolved a strict specificity for phosphoserine sites followed immediately by the sequence Pro-X-Lys/Arg (with strong preference for Lys in most studied orthologs), making it functionally a phosphoserine phosphatase (35, 36). This substrate specificity appears invariant across the Dikarya subkingdom that contains the major plant and animal fungal pathogens (29), implying inhibitors could have broad applicability against fungal diseases. The structural basis for Cdc14’s specificity is known, with tandem DSP-like folds creating a unique peptide binding groove at their interface, adjacent to the catalytic pocket (37, 38). This domain structure is a signature feature of the Cdc14 family (29) and the active site specificity it imparts suggests development of highly selective Cdc14 inhibitors could be achievable. Indeed, highly specific small molecule inhibitors of human Cdc14A and Cdc14B were recently reported (39).

In addition to the conserved tandem DSP domains responsible for substrate recognition and catalysis, Cdc14 enzymes contain a poorly conserved C-terminal region of predicted disorder and varying length. The DSP domains alone are sufficient to perform the essential function in *S. cerevisiae*, however an early study observed that they exhibit a reduced catalytic rate compared to full-length Cdc14 (2) and it was later found that the essential function in *S. cerevisiae* requires only a small fraction of normal Cdc14 activity (40). A similar difference in activity was observed between full-length and truncated forms of the Cdc14 ortholog from *F. graminearum* (22), suggesting that the C-terminal tail makes some conserved contribution to enzyme activity. In support of this, studies in *S. cerevisiae* and *S. pombe* reported inhibitory effects of phosphorylation sites in the C-terminal tail on enzyme activity, further suggesting that the tail region may be a target for posttranslational regulation of Cdc14 enzyme activity (41, 42). However, the mechanism by which the putatively disordered C-terminal tail might influence Cdc14 catalytic rate is unknown.

We recently identified a short, conserved motif in the disordered C-terminal region of fungal Cdc14 orthologs (Fig. 1*A*) responsible for the observed differences in catalytic rate between full-length enzymes and catalytic domain truncations (28). This motif is functionally important, as point mutations in *C. albicans* Cdc14 resulted in hypersensitivity to cell wall stress, defective cell separation and hyphal development, and loss of virulence (28). The core sequence of the motif, Gln-Pro-Arg-Lys, resembles the optimal Cdc14 substrate recognition sequence, with glutamine in place of a substrate phosphoserine (Fig. 1*B*), raising the possibility that it acts as a pseudosubstrate that binds the Cdc14 active site. Although invariant in Dikarya Cdc14 orthologs, this sequence is not found in metazoan Cdc14s, suggesting it might reflect a mechanistic difference between these major eukaryotic lineages. This prompted us to characterize the biochemical mechanism by which this conserved fungal motif contributes to normal catalysis and further explore if this mechanism is indeed unique to fungal Cdc14 enzymes. Our results support a mechanistic model where this motif binds the active site at the phosphoenzyme stage of the catalytic cycle to stimulate the rate-limiting hydrolysis step, representing a novel feature of the Cdc14 catalytic mechanism. We named the motif “substrate-like catalytic enhancer” (SLiCE) to reflect this. We provide evidence that the SLiCE motif is a target for *in vivo* regulation of Cdc14 activity. Surprisingly, we found that vertebrate Cdc14 enzymes evolved a distinct, but functionally analogous, motif. Importantly, the fungal and human pseudosubstrate motifs were not interchangeable, providing the first evidence of structural and/or mechanistic differences in their active sites that supports the possibility of developing fungal-specific Cdc14 inhibitors.

**Figure 1:**
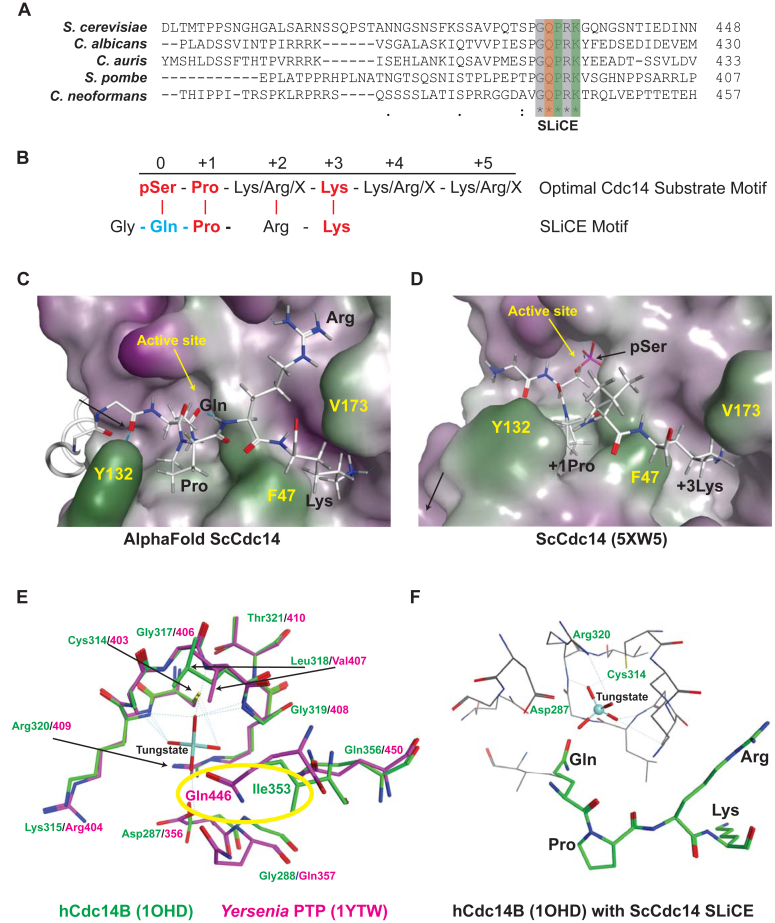
**The highly conserved fungal SLiCE motif is predicted to bind the Cdc14 active site.***A*, multiple sequence alignment showing a portion of the C-terminal tail of Cdc14 orthologs from several model and pathogen fungal species generated with Clustal Omega. The invariant SLiCE motif is highlighted. *Asterisks* indicate identical amino acids in all aligned sequences. *Orange* and *green highlighting* indicates the Gln and Pro + Lys residues subjected to mutagenesis, respectively. *B*, comparison of the known optimal Cdc14 substrate motif with the fungal SLiCE motif. *Red text* indicates amino acids essential for efficient substrate recognition by Cdc14 enzymes (35, 36). *Blue* indicates the Gln predicted to facilitate coordination of nucleophilic water for phosphoenzyme hydrolysis. *C*–*D*, AlphaFold2 structural prediction of ScCdc14 showing the active site region with bound SLiCE motif compared to same view of the ScCdc14 crystal structure bound to an optimal phosphopeptide substrate (RCSB: 5XW5). The catalytic domain is depicted in surface representation (*green* = nonpolar, *purple* = polar) and the SLiCE motif and phosphopeptide substrate in *stick mode*. The active site and several amino acids important for substrate binding are labeled in *yellow* for orientation. SLiCE motif residues and key substrate recognition positions are labeled in *black*. *E*, structures of hCdc14B (RCSB: 1OHD) and *Yersinia* PTP (RCSB: 1YTW), each bound to the PTP inhibitor tungstate, were superposed in Molecular Operating Environment software. The active site region is shown (tungstate from 1OHD was removed for simplicity) with hCdc14B backbone and residue labels in *green* and *Yersinia* PTP in *magenta*. The critical water-coordinating Gln of *Yersinia* PTP that is absent from Cdc14 enzymes is highlighted with *yellow oval*. *F*, the AlphaFold2 ScCdc14 structure was superposed on hCdc14B (1OHD) in Molecular Operating Environment. Relative position of the ScCdc14 SLiCE motif Gln to the bound tungstate in the hCdc14B active site is shown. SLiCE sequence is the *thick green stick model* with *black residue labels*. Critical catalytic residues of hCdc14B are labeled in *green*. SLiCE, substrate-like catalytic enhancer; PTP, protein tyrosine phosphatase.

## Results

### The fungal SLiCE motif binds the Cdc14 active site in AlphaFold2 structure models

Structural data for Cdc14 enzymes is restricted to the conserved catalytic domain (37, 38), and thus we predicted the structure of full-length Cdc14 from *S. cerevisiae* (ScCdc14) and various other fungal species using AlphaFold2 (43) to learn about potential structural features in the C terminus, including the SLiCE motif. In the predicted structures of Cdc14 orthologs from the model yeasts *S. cerevisiae* and *S. pombe* and the pathogenic yeasts *C. albicans* and *Candida auris*, the C-terminal tail region is disordered, as expected; however, in each case the SLiCE motif contacts the catalytic domain in the active site region (Fig. S1). Similar active site binding of the SLiCE motif was observed in AlphaFold2 structure predictions of Cdc14 orthologs from several other fungal species as well (not shown). The Pro and Lys of the SLiCE motif make contacts equivalent to the substrate +1 Pro and +3 Lys in the ScCdc14 crystal structure, 5XW5 ((38); Fig. 1, *C* and *D*). The Gln of the SLiCE motif occupies a portion of the catalytic pocket occupied by the substrate phosphoSer. The position of the SLiCE Gln is reminiscent of an active site Gln in PTPs that coordinates the attacking water molecule during the phosphoenzyme hydrolysis step (44, 45, 46). A comparison of the crystal structures of hCdc14B and the *Yersenia pestis* PTP bound to the inhibitor tungstate, which mimics the phosphoenzyme transition state, revealed that Cdc14 enzymes lack a Gln in a similar position within the active site (Fig. 1*E*). Instead, an Ile that helps form the hydrophobic pocket for the substrate +1 Pro sidechain (37) sits in this position. An overlay of the ScCdc14 SLiCE motif with the tungstate-bound hCdc14B structure suggests that the invariant SLiCE Gln could be in position to help coordinate the nucleophilic water, similar to the active site Gln of classical PTPs (Fig. 1*F*).

### The SLiCE motif enhances the rate-limiting Cdc14 catalytic step

**Figure 2:**
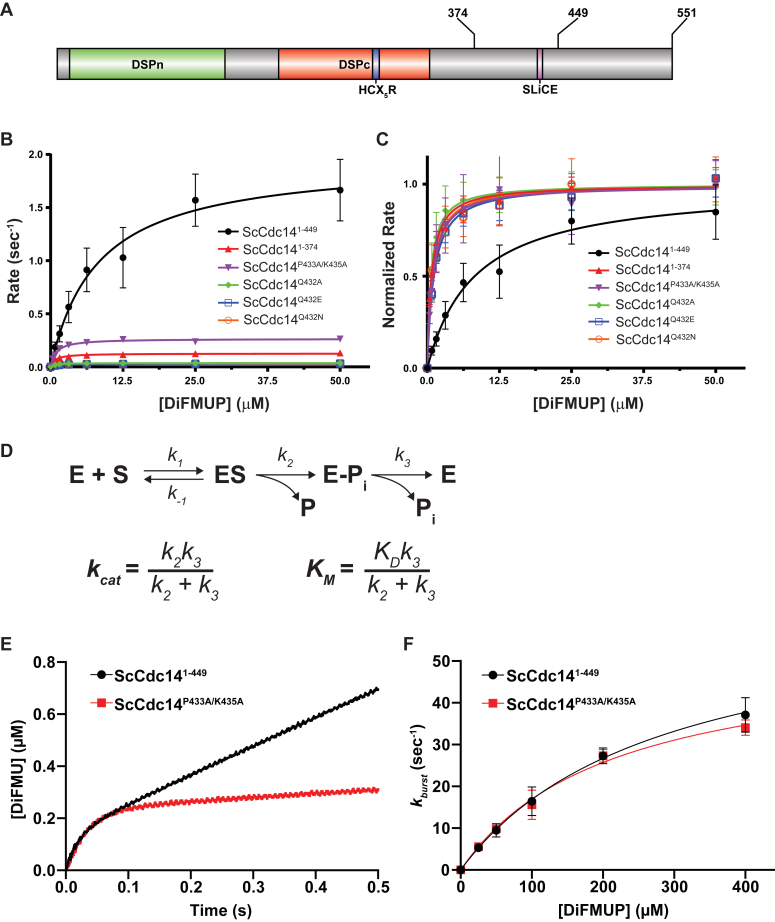
**The SLiCE motif enhances the rate-limiting Cdc14 catalytic step.***A*, domain map of ScCdc14. DSPn and DSPc are the N- and C-terminal dual-specificity phosphatase domains, respectively. HCX_5_R is the signature PTP phosphate-binding loop motif with catalytic Cys. Sites of truncations and the invariant fungal SLiCE motif are indicated. *B*, initial velocities as a function of increasing DiFMUP concentration were measured under steady-state conditions for the indicated ScCdc14 variants. All point mutations were made in ScCdc14^1-449^. Data are average values from at least three independent measurements. Error bars are SDs. *Lines* are best fits for the Michaelis–Menten equation. Resulting kinetic parameters *k*_*cat*_ and *K*_*M*_ are listed in Table S1. *C*, to better visualize differences in *K*_*M*_, all *V*_*max*_ values from data in panel A were normalized to 1. *D*, *top:* the general PTP-catalyzed reaction scheme with associated rate constants (50), where E is enzyme, S is phosphorylated substrate, P is dephosphorylated product, P_i_ is inorganic phosphate, and E-P_i_ is the covalent phosphoenzyme intermediate. For simplicity, the reverse catalytic steps are omitted and considered negligible under our experimental conditions. *Bottom:* definitions of *k*_*cat*_ and *K*_*M*_ for enzymes following the two step PTP reaction mechanism (47). *E*, a representative presteady-state reaction progress graph for reaction of the indicated ScCdc14 enzyme variants with 200 μM DiFMUP, measured in a stopped flow spectrofluorometer. Data were fit with Equation 1 (see Experimental procedures) to generate rates of the burst phase (*k*_*burst*_) of the reaction. *F*, *k*_*burst*_ was plotted as a function of substrate concentration from presteady-state kinetic measurements with the substrate DiFMUP and the indicated ScCdc14 enzyme variants. Data are averages of three independent measurements and error bars are SDs. Data were fit in GraphPad Prism with Equation 2 (Experimental procedures). DiFMUP, 6,8-difluoro-4-methylumbelliferyl phosphate; PTP, protein tyrosine phosphatase; SLiCE, substrate-like catalytic enhancer.

To experimentally characterize the SLiCE motif mechanism, we expressed and purified several recombinant ScCdc14 enzyme variants (Figs. 2*A* and S2*A*). These included a truncation (ScCdc14^1-449^) that retains the SLiCE motif and exhibits activity indistinguishable from full-length ScCdc14 (28) and the catalytic domain alone lacking the SLiCE motif (ScCdc14^1-374^). We also purified the following SLiCE motif point mutants generated in ScCdc14^1-449^: ScCdc14^P433A/K435A^ (predicted to severely reduce active site binding) and ScCdc14^Q432A^, ScCdc14^Q432E^, and ScCdc14^Q432N^ (predicted to impair hydrolytic water coordination). We first compared the ScCdc14 variants under steady-state conditions using the fluorescent small molecule substrate 6,8-difluoro-4-methylumbelliferyl phosphate (DiFMUP). Consistent with our previous report using *p*-nitrophenyl phosphate and phosphopeptide substrates (28), ScCdc14^1-374^ displayed a 16-fold decrease in *k*_*cat*_ compared to ScCdc14^1-449^ (Fig. 2*B*, Table S1). All ScCdc14^1-449^ point mutants also displayed significant decreases in *k*_*cat*_, confirming that these residues are required for the SLiCE catalytic contribution. The effect of the P433A/K435A mutations on *k*_*cat*_ was less than mutations of Q432, suggesting that some SLiCE function may persist in the absence of these amino acids. Interestingly, in addition to decreased *k*_*cat*_, ScCdc14^1-374^ and all SLiCE point mutants also exhibited similar magnitude (8- to 12-fold) decreases in *K*_*M*_ (Fig. 2*C*, Table S1). Considering the definitions of *k*_*cat*_ and *K*_*M*_ for the two-step PTP catalytic mechanism (Fig. 2*D*; (47)), only an effect on the rate constant *k*_*3*_ for the phosphoenzyme hydrolysis step would cause similar changes in both parameters. To test if the SLiCE motif specifically impacts phosphoenzyme hydrolysis, we performed presteady-state kinetics on a stopped flow instrument comparing ScCdc14^1-449^ and ScCdc14^P433A/K435A^ using a previously established assay (34). Both enzymes showed similar burst phases over a range of substrate concentrations (Fig. 2, *E* and *F*). The burst is largely governed by binding equilibrium (*K*_*D*_) and the first enzymatic step (*k*_*2*_) and suggests that the SLiCE motif does not significantly influence these phases of the Cdc14 catalytic cycle. Conversely, the postburst linear rate of ScCdc14^P433A/K435A^, primarily reflecting the rate-limiting *k*_*3*_, was greatly reduced compared to ScCdc14^1-449^ (Fig. 2*E*), similar to the reduction in *k*_*cat*_. We conclude that the SLiCE motif functions primarily to accelerate the rate-limiting phosphoenzyme hydrolysis step. Moreover, the strict requirement for glutamine at position 432 is consistent with a role in coordinating the hydrolytic water similar to Gln446 in the catalytic domain of *Yersinia* PTP (46).

### The SLiCE motif functionally interacts with the active site

**Figure 3:**
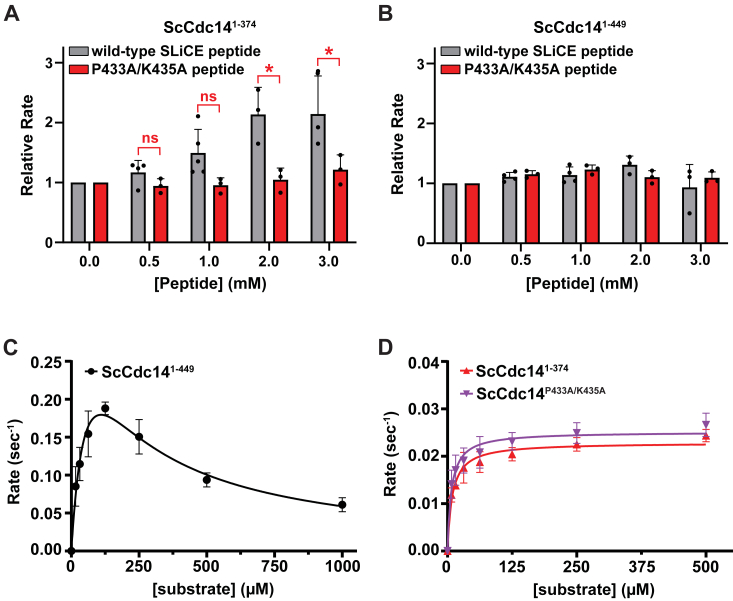
**The SLiCE motif functionally interacts with the Cdc14 active site.***A*, the ability of synthetic peptides containing WT or mutant SLiCE sequence to stimulate reaction rate of the ScCdc14^1-374^ catalytic domain (lacking the SLiCE motif) was measured under the same steady-state conditions used in Figure 2 with near-saturating DiFMUP concentration (60 μM). WT SLiCE peptide = QTSPGQPRKGQN; P433A/K435A peptide = QTSPGQARAGQN. Rates are normalized values relative to reactions with no added peptide. Data are averages of at least three independent measurements and error bars are SDs. ∗ = *p* value <0.05 in two-way ANOVA with Tukey post hoc test comparing the WT and P433A/K435A peptides at each concentration; ns = not significant (*p* > 0.05). *B*, same as panel A with ScCdc14^1-449^ containing the native SLiCE motif. All *p* values were >0.05. *C*–*D*, steady-state kinetic analyses with the indicated ScCdc14 enzyme variants toward an optimal phosphopeptide substrate derived from the *Saccharomyces cerevisiae* Acm1 protein (sequence = MI(pS)PSKKRTIL, where pS is phosphoSer). Data are averages of three independent measurements and error bars are SDs. *Lines* were generated by fitting data with a modified Michaelis–Menten equation containing a substrate inhibition term in Prism. DiFMUP, 6,8-difluoro-4-methylumbelliferyl phosphate; SLiCE, substrate-like catalytic enhancer.

Although AlphaFold2 reproducibly predicted SLiCE motif binding to the Cdc14 active site, we were unable to measure binding of synthetic peptides containing the SLiCE sequence to ScCdc14^1-374^, suggesting affinity for the native enzyme is very low (not shown). We speculated that the motif might selectively interact with the phosphoenzyme intermediate. We do not have a way to isolate stable phosphoenzyme, and therefore turned to kinetic experiments to test if the SLiCE motif interacts with the active site during catalysis. We first tested if synthetic peptides representing WT and mutant SLiCE sequences could stimulate activity of ScCdc14^1-449^ and ScCdc14^1-374^ *in trans*. The catalytic rate of ScCdc14^1-374^, which lacks the SLiCE motif, exhibited dose-dependent stimulation by a peptide with WT SLiCE sequence, but not by a variant with the P433A and K435A substitutions predicted to disrupt active site binding (Fig. 3*A*). In contrast, the catalytic rate of ScCdc14^1-449^, which retains the SLiCE motif, was not further stimulated by either synthetic peptide (Fig. 3*B*). These data demonstrate that the SLiCE motif can physically interact with the Cdc14 catalytic domain to enhance catalytic rate and this interaction requires the Pro-X-Lys sequence common to Cdc14 substrates.

Previously, we observed strong substrate inhibition of ScCdc14 in reactions with high affinity phosphopeptide substrates, an observation we were unable to explain at the time (35). Based on our new SLiCE motif observations, we hypothesized that this substrate inhibition could be due to binding competition between an incoming substrate and the SLiCE motif at the phosphoenzyme stage of the catalytic cycle. To test this, we compared the steady-state kinetics of ScCdc14^1-374^ and ScCdc14^P433A/K435A^ (both lacking a functional SLiCE motif) to ScCdc14^1-449^ (retaining the SLiCE motif) with an optimal phosphopeptide substrate. If our hypothesis were correct, ScCdc14^1-374^ and ScCdc14^P433A/K435A^ would not exhibit substrate inhibition because they lack a functional SLiCE motif for substrates to compete with. ScCdc14^1-449^ showed clear substrate inhibition as previously reported (Fig. 3*C*), whereas ScCdc14^1-374^ and ScCdc14^P433A/K435A^ produced normal hyperbolic velocity *versus* substrate curves, even at substrate concentrations 50-fold higher than *K*_*M*_ (Fig. 3*D*). The declining initial velocity of ScCdc14^1-449^ at high substrate approached the maximal velocity of ScCdc14^1-374^ and ScCdc14^P433A/K435A^. This kinetic result provides strong evidence that the SLiCE motif binds to the Cdc14 active site as a pseudosubstrate, preferentially at the phosphoenzyme step of the catalytic cycle.

### The SLiCE motif does not affect binding of good substrates but discriminates against poor substrates

**Figure 4:**
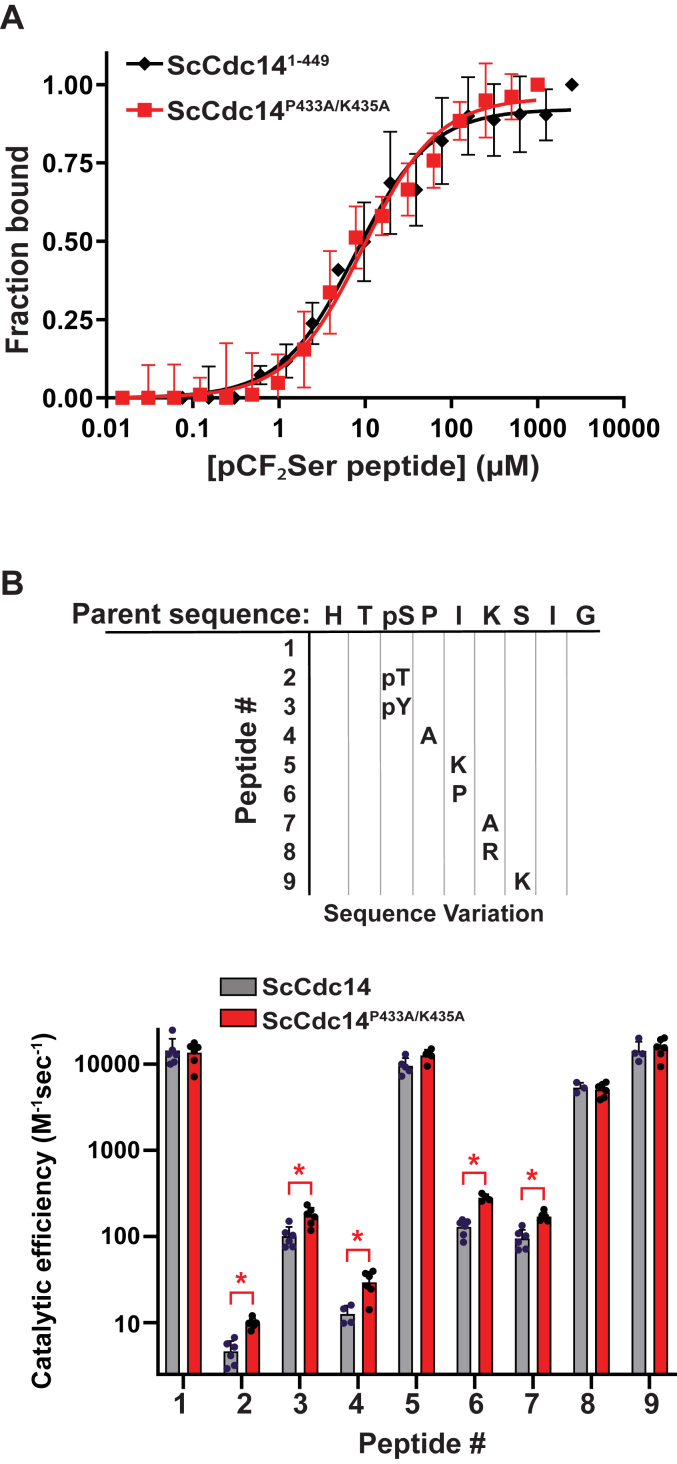
**The SLiCE motif does not affect high affinity substrate binding but helps discriminate against poor substrates *in vitro*.***A*, binding of a phosphopeptide substrate analog containing a noncleavable pCF_2_Ser residue to the indicated ScCdc14 enzyme variants was measured by microscale thermophoresis. Data are the average of three independent measurements and error bars are SDs. Data were fit with a standard dose-response function in GraphPad Prism to extract the equilibrium dissociation constant (*K*_*D*_). *B*, catalytic efficiencies (*k*_*cat*_*/K*_*M*_) of the indicated Cdc14 enzyme variants were measured on a pooled collection of phosphopeptides of varying sequence using LC-MS detection. The *chart at top* illustrates the sequence variations represented in the pool, starting with an optimal substrate sequence (HT(pS)PIKSIG) derived from the *Saccharomyces cerevisiae* Yen1 protein (36). Each variant has one position known to influence substrate recognition altered to a different amino acid. pS, pT, and pY represent phosphorylated Ser, Thr, and Tyr amino acids. *k*_*cat*_*/K*_*M*_ values in the *bar graph at bottom* are averages of at least four independent measurements with SD error bars. ∗ = *p* value <0.05 after analysis by multiple unpaired *t* test with individual variance in Prism comparing mean *k*_*cat*_*/K*_*M*_ of ScCdc14^1-449^ and ScCdc14^P433A/K435A^ for each phosphopeptide substrate (otherwise *p* > 0.05). SLiCE, substrate-like catalytic enhancer.

To explore if the SLiCE motif influences initial substrate binding, we measured the dissociation constant, *K*_*D*_, of an optimal peptide substrate analog containing a noncleavable phosphonoserine residue (29, 48) with ScCdc14^1-449^ and ScCdc14^P433A/K435A^ (Fig. 4*A*). The *K*_*D*_ values were very similar (6 ± 2 and 13 ± 4 μM for ScCdc14^1-449^ and ScCdc14^P433A/K435A^, respectively) and consistent with the *K*_*i*_ measured for this peptide previously (29). Thus, the SLiCE motif appears to have negligible impact on binding of high affinity substrates, consistent with its apparently weak affinity for the native enzyme and with the idea that it binds preferentially to the phosphoenzyme. To test if the SLiCE motif might affect binding to low affinity substrates, we measured the catalytic efficiencies (*k*_*cat*_*/K*_*M*_) of a collection of phosphopeptides with sequence variations known to influence recognition by Cdc14 enzymes (35, 36). We used very low substrate concentrations and mass spectrometry for product detection as described previously (29, 49). At substrate concentrations well below *K*_*M*_ formation of enzyme substrate becomes rate-limiting and reaction velocity is a useful measure of substrate binding affinity. Consistent with the binding results, the catalytic efficiencies of ScCdc14^1-449^ and ScCdc14^P433A/K435A^ for high affinity substrates were indistinguishable (Fig. 4*B*). However, for low affinity substrates, we observed a modest, but consistent and statistically significant, increase in catalytic efficiency for the ScCdc14^P433A/K435A^ enzyme lacking a functional SLiCE motif. This suggests that while the SLiCE motif does not impact binding of good substrates, it does weakly bind the native active site and may function in part to enhance Cdc14 substrate selectivity by discriminating against poor substrates.

### Vertebrate Cdc14 enzymes have a distinct, but mechanistically similar, SLiCE motif

**Figure 5:**
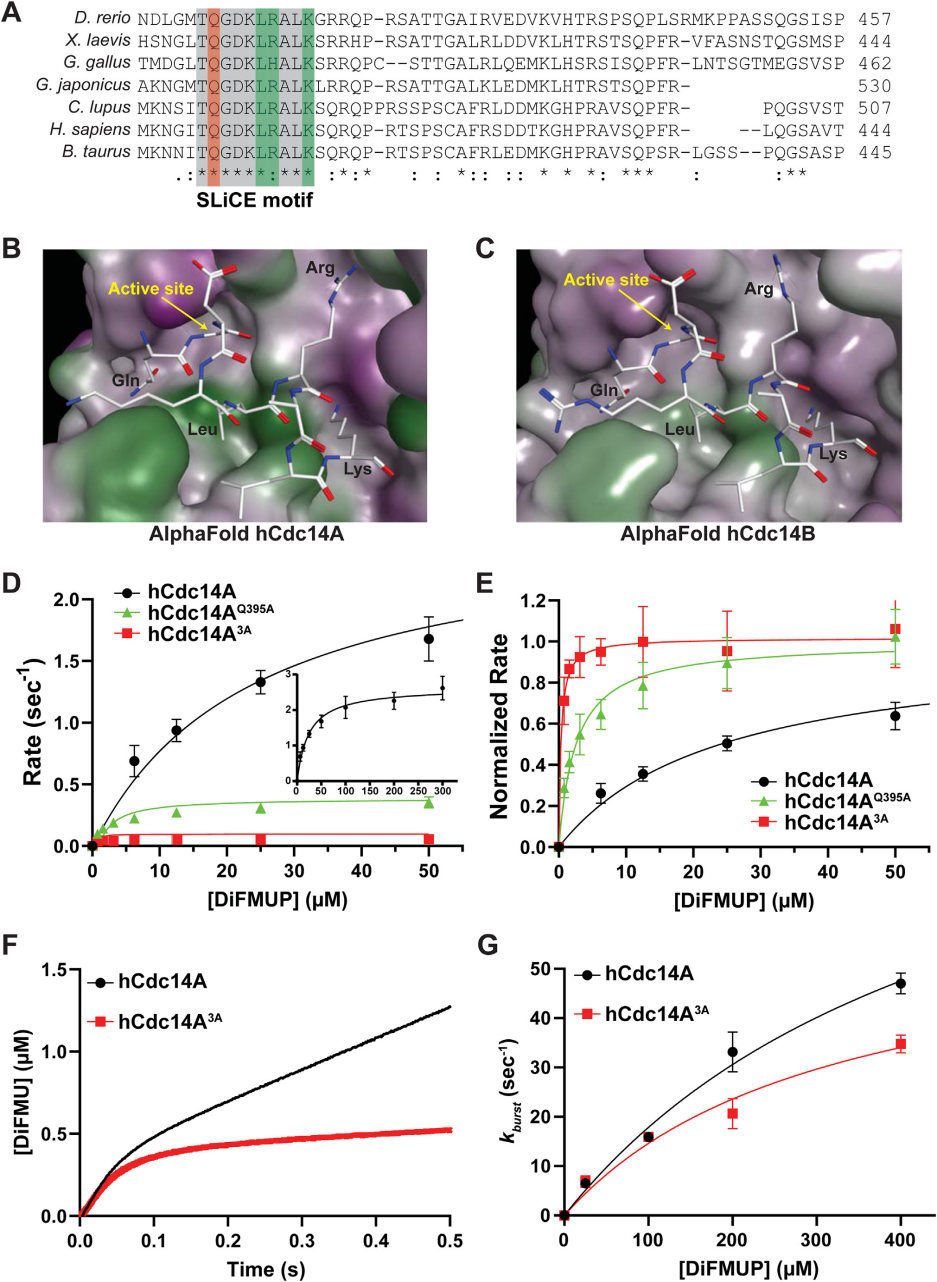
**Vertebrate Cdc14 enzymes have a distinct, but mechanistically similar, SLiCE motif.***A*, similar to Figure 1*A*, multiple sequence alignment showing a portion of the C-terminal tail of Cdc14A orthologs from representative vertebrate species generated in Clustal Omega. SLiCE motif is boxed in *gray*. The Gln predicted to help coordinate the nucleophilic water is highlighted in *orange* and residues predicted to contribute significantly to active site binding are highlighted in *green*. *B*–*C*, active site region of AlphaFold2 structure predictions for hCdc14A and hCdc14B. The conserved catalytic domain is depicted in surface rendering. The putative SLiCE motif is depicted with *stick mode* and residues predicted to be important for SLiCE function are labeled. *D*–*E*, steady-state kinetic analyses with hCdc14A enzyme variants toward DiFMUP as in Figure 2, *B* and *C*. Inset in panel D shows the full curve for WT hCdc14A. In E, *V*_*max*_ values from data in panel D were normalized to 1 to show differences in *K*_*M*_. *F*–*G*, presteady-state kinetic analysis of the indicated hCdc14A variants with 100 μM DiFMUP as in Figure 2, *E* and *F*. DiFMUP, 6,8-difluoro-4-methylumbelliferyl phosphate; SLiCE, substrate-like catalytic enhancer.

The uniqueness of the SLiCE sequence to fungal Cdc14s could reflect a mechanistic difference between fungal and nonfungal Cdc14 enzymes. To explore if human Cdc14 enzymes might have a similar pseudosubstrate motif, we predicted the structures of hCdc14A and hCdc14B with AlphaFold2. To our surprise, the AlphaFold2 structures of hCdc14A and hCdc14B both had identical C-terminal sequences docked in their active sites (Fig. S3, *A*–*B*), similar to the fungal Cdc14 structures. Unlike the fungal SLiCE motif, which lacks secondary structure, the docked sequence in hCdc14A and hCdc14B is an alpha helix and does not share obvious similarity to the optimal Cdc14 substrate motif. The sequence is highly conserved across vertebrates, despite the poor overall conservation of the C-terminal region (Fig. 5*A*), and AlphaFold2 predictions of other vertebrate Cdc14 structures invariably had this alpha helix positioned in the active site (not shown). Inspection of the binding mode of this motif revealed structural similarities to the fungal SLiCE motif that were not obvious from the primary sequence (Fig. 5, *B* and *C*). An invariant Gln is also positioned in the active site pocket, the hydrophobic pocket that accommodates the substrate +1 Pro is occupied by the nonpolar side chain of a Leu, and Arg and Lys residues occupy the same relative positions as the fungal SLiCE Arg and Lys. We therefore hypothesized that this motif acts similar to the fungal SLiCE motif to stimulate catalysis. We tested this by expressing and purifying hCdc14A variants (Fig. S2*B*) with point mutations ablating either the glutamine (Q395A) or residues predicted to mimic substrate binding features (L399A, R400A, and K403A—henceforth called “3A”) and compared their activity to WT hCdc14A. In steady-state assays with DiFMUP, both hCdc14^Q395A^ and hCdc14A^3A^ displayed approximately equivalent decreases in both *k*_*cat*_ and *K*_*M*_ compared to WT hCdc14A, with the effect of the 3A mutation slightly more severe (Fig. 5 *D* and *E*, Table S1). The same analysis was performed with hCdc14B, with similar results (Fig. S3, *C*–*D*, Table S1). We next measured WT hCdc14A and hCdc14A^3A^-catalyzed reactions under presteady-state conditions. Similar to the results observed with ScCdc14, the major difference between the WT and mutant hCdc14A enzymes was the linear phase (Fig. 5*F*), reflecting primarily *k*_*3*_, although a small difference in the burst phase was also observed at high substrate concentrations (Fig. 5*G*).

**Figure 6:**
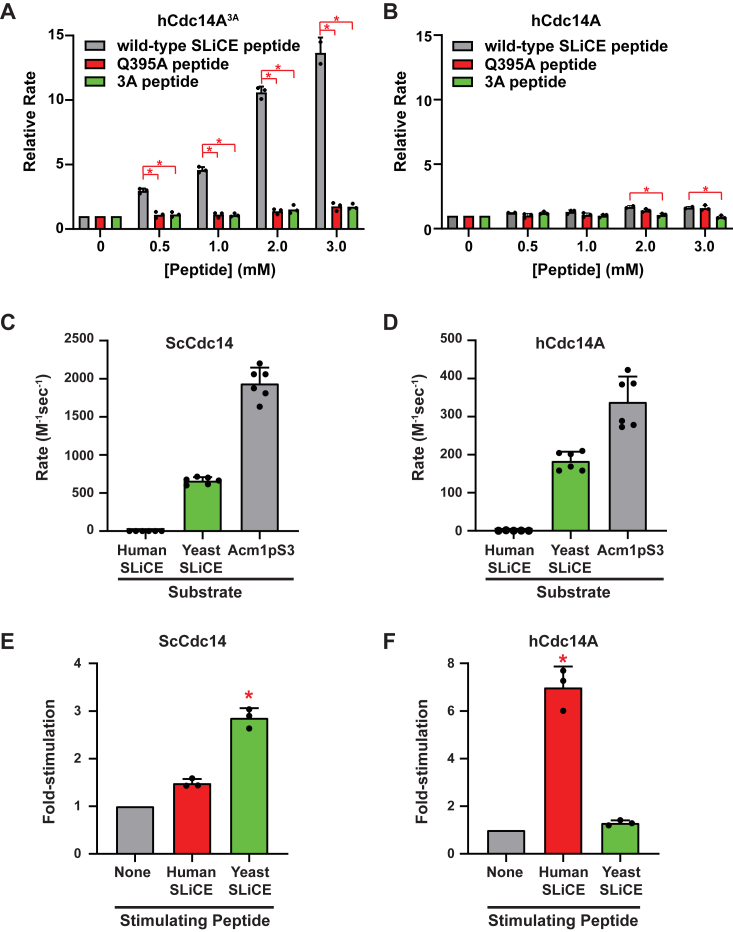
**The human SLiCE motif stimulates the hCdc14A catalytic domain *in trans* but is not functionally interchangeable with the fungal SLiCE motif.***A*, the ability of synthetic peptides containing WT or mutant human SLiCE sequence to stimulate reaction rate of the hCdc14A^3A^ mutant lacking a functional SLiCE motif was measured under steady-state conditions with near-saturating DiFMUP concentration (300 μM) as in Figure 3*A*. WT SLiCE peptide = TQGDKLRALKSQR; Q395A peptide = TAGDKLRALKSQR; 3A peptide = TQGDKAAALASQR. *B*, same as panel A with WT hCdc14A that contains a functional SLiCE motif. In both A and B, ∗ = *p* value <0.05 in two-way ANOVA with Tukey post hoc test comparing stimulation by WT peptide to Q395A and 3A peptides at each concentration; otherwise *p* > 0.05. *C*, dephosphorylation of phosphopeptide derivatives of the *Saccharomyces cerevisiae* (yeast) and human Cdc14A SLiCE motif sequences by ScCdc14^1-449^ were compared to that of an optimal Acm1pS3 phosphopeptide substrate. The human SLiCE peptide sequence was T(pS)GDKLRALKSQR, and the yeast SLiCE peptide sequence was QTSPG(pS)PRKGQN where pS is phosphoserine. All reactions contained 200 μM peptide substrate and were run for 30 min. Enzyme concentrations varied: 1 μM for reactions with the human SLiCE substrate, 50 nM for ScCdc14^1-449^ with the yeast SLiCE substrate, and 25 nM for ScCdc14^1-449^ with the Acm1pS3 substrate. Data are means of six measurements and error bars are SDs. *D*, same as panel C with hCdc14A enzyme at 1 μM for human SLiCE substrate and 100 nM for yeast SLiCE and Acm1pS3 substrates. *E*, peptide stimulation assays using 10 μM DiFMUP as substrate were conducted as in Figures 3*A* and 6*A* with ScCdc14^1-374^ (50 nM) and 2 mM each stimulating peptide. *F*, same as panel E with hCdc14A^3A^ (10 nM) using 100 μM DiFMUP. In E and F, differences between the unnormalized means of the three datasets were assessed by one way ANOVA with Tukey post hoc test. ∗ = *p* value <0.05 comparing human or yeast SLiCE peptide stimulation to the unstimulated value; otherwise *p* > 0.05. DiFMUP, 6,8-difluoro-4-methylumbelliferyl phosphate; SLiCE, substrate-like catalytic enhancer.

A synthetic peptide representing the vertebrate SLiCE motif sequence strongly stimulated activity of hCdc14A^3A^ *in trans* but had a relatively small effect on WT hCdc14A activity (Fig. 6, *A* and *B*). Peptides variants with either the Q395A or 3A substitutions had little effect on activity of either enzyme, again consistent with ScCdc14 results. Finally, we attempted to compare substrate inhibition with WT and mutant hCdc14A like we did for ScCdc14. Surprisingly, hCdc14A did not exhibit any evidence of substrate inhibition with the same phosphopeptide substrate used in the corresponding ScCdc14 experiment in Figure 3*C* (not shown). This difference may reflect a divergence in the catalytic mechanism of fungal and vertebrate Cdc14s that could be related to the distinct SLiCE motif structures (see below and Discussion). Nonetheless, the structural predictions and kinetic analyses show that vertebrate Cdc14 enzymes also have a SLiCE motif that acts to stimulate phosphoenzyme hydrolysis.

### The fungal and vertebrate SLiCE motifs are not functionally interchangeable

Not surprisingly, replacing the invariant Gln of the fungal SLiCE motif with a phosphoSer creates an excellent Cdc14 substrate for both ScCdc14 and hCdc14A, almost as reactive as the optimal Acm1pS3 phosphopeptide (Fig. 6, *C* and *D*). We wondered if the vertebrate SLiCE sequence could similarly be converted into an efficient substrate by replacing its Gln with phosphoSer. If so, it could mean that Cdc14 enzymes might recognize a previously unappreciated class of substrates. However, the phosphoSer-containing human SLiCE peptide could not be dephosphorylated by either ScCdc14 or hCdc14A enzymes, even at high enzyme concentration. The more rigid alpha helical structure of this motif may not allow proper engagement of the phosphate moiety with the catalytic pocket or the addition of phosphoSer could disrupt the helical secondary structure needed for binding. Finally, we used the peptide *trans* stimulation assays to test if the distinct fungal and vertebrate SLiCE motifs are functionally interchangeable. Interestingly, the yeast SLiCE peptide was only able to significantly stimulate ScCdc14, exerting no effect on hCdc14A activity under identical conditions (Fig. 6, *E* and *F*), even though the phosphorylated form of this peptide was a good hCdc14A substrate. Similarly, the human SLiCE peptide failed to significantly stimulate ScCdc14 despite strongly stimulating hCdc14A. Like the substrate inhibition effect, these results argue for some difference in active site conformation at the phosphoenzyme stage between fungal and vertebrate Cdc14s, despite their nearly identical native active site structures and substrate recognition motifs.

### The fungal SLiCE motif is regulated by phosphorylation

**Figure 7:**
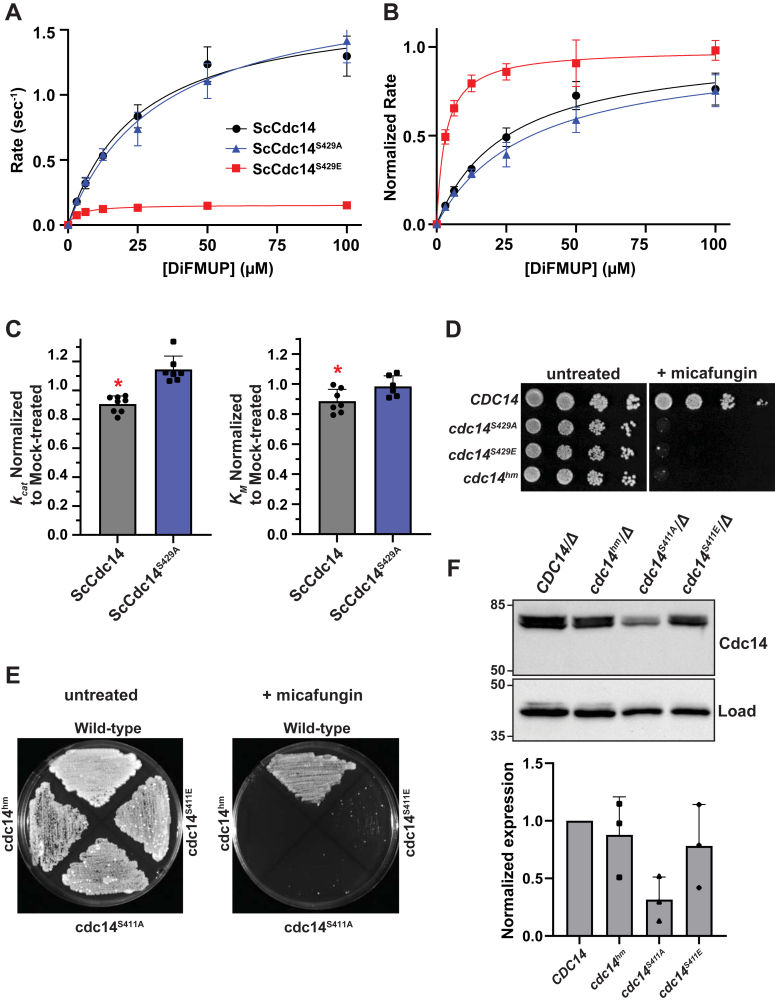
**The fungal SLiCE motif is regulated by phosphorylation.***A*–*B*, steady-state kinetic analyses with ScCdc14 enzyme variants containing Ala or Glu substitutions in the S429 Cdk phosphorylation site, as in Figure 2, *A* and *B*. *C*, *k*_*cat*_ and *K*_*M*_ measurements of ScCdc14 and the ScCdc14^S429A^ phosphosite mutant after 16 h treatment with affinity-purified Cdk1 (Clb2-Cdc28) and ATP. *k*_*cat*_ and *K*_*M*_ were normalized to values obtained from mock-treated enzymes (see Experimental procedures). Values are means of at least six independent measurements and error bars are SDs. Unpaired *t*-tests assuming equal variance (two-tailed) were used to determine statistical significance of mock-normalized *k*_*cat*_ and *K*_*M*_ measurement differences between Cdk1-treated ScCdc14 and ScCdc14^S429A^; ∗ = *p* value < 0.05. *D*, agar plate spotting assay with identical serial dilutions of liquid cultures of the indicated *Saccharomyces cerevisiae* strain genotypes on YPD or YPD + 20 ng/ml micafungin. Plates were incubated at 30 °C for 48 h (untreated) or 72 h (+micafungin) prior to imaging. *E*, agar plate patch assay with the indicated *Candida albicans* strains on YPD or YPD + 50 ng/ml micafungin. Plates were incubated at 30 °C for 72 h prior to imaging. Images in panels D and E are representative of multiple experiments performed with three independent isolates of the Ala and Glu mutant strains. *F*, *top:* immunoblotting of Cdc14-3xHA in cell extracts from log phase YPD liquid cultures of the indicated strains probed with anti-HA and anti-PSTAIR (load control) antibodies. Numbers at *left* are size markers, in kDa. *Bottom:* quantitation of blots from three independent experiments. Bars are means with SDs. Expression levels are normalized first to the load control and then to the WT *CDC14* strains. Means were compared by one way ANOVA with Tukey post hoc test. All *p* values were >0.05. Cdk, cyclin-dependent kinase; HA, hemagluttinin; SLiCE, substrate-like catalytic enhancer; YPD, yeast extract/peptone/dextrose.

The broad conservation of both fungal and vertebrate SLiCE motifs suggests that they are biologically important. Indeed, we recently reported that point mutations in this motif in *S. cerevisiae* and *C. albicans CDC14* causes hypersensitivity to cell wall stress and in *C. albicans* also impairs hyphal development and virulence (28). An earlier report suggested that phosphorylation of a conserved Ser-Pro site immediately preceding the fungal SLiCE motif (Fig. 1*A*) by Cdk1 decreases ScCdc14 activity *in vitro* and *in vivo* (41). A similar result was reported for a collection of phosphorylation sites that included the homologous Thr-Pro Cdk site in the *S. pombe* Cdc14 ortholog, Clp1 (42). These results suggest that Cdk phosphorylation could regulate Cdc14 activity by modulating SLiCE motif function. To test this, we expressed and purified ScCdc14 enzymes with phospho-ablating (ScCdc14^S429A^) and phospho-mimetic (ScCdc14^S429E^) mutations (Fig. S2*A*) and measured their kinetic parameters in DiFMUP steady-state assays. While ScCdc14^S429A^ and ScCdc14^1-449^ exhibited nearly identical kinetics, the ScCdc14^S429E^ enzyme displayed a significant decrease in both *k*_*cat*_ and *K*_*M*_ (Fig. 7, *A* and *B*), consistent with the trends observed with SLiCE motif mutations and with the hypothesis that phosphorylation at this residue inhibits SLiCE motif function. To directly test if phosphorylation impacts SLiCE motif function, we immunoaffinity purified Cdk1 (Clb2-Cdc28) from *S. cerevisiae* cells and treated WT ScCdc14 and ScCdc14^S429A^ enzymes with it. After removing the Cdk1-bound affinity resin, we measured effects on Cdc14 steady-state kinetic parameters with DiFMUP. We note that Cdc14 likely can autodephosphorylate this Ser-Pro site at a slow rate, potentially obscuring any effects. Nonetheless, treatment of WT ScCdc14 with Cdk1 beads caused an approximately equivalent, and statistically significant, decrease in *k*_*cat*_ and *K*_*M*_ relative to mock-treated ScCdc14 (Fig. 7*C*). In contrast, *K*_*M*_ for ScCdc14^S429A^ was indistinguishable between the Cdk1- and mock-treated samples and *k*_*cat*_ actually increased slightly after Cdk1 treatment. These results support the idea that phosphorylation of Ser429 by Cdk1 suppresses Cdc14 activity by inhibiting SLiCE motif function.

To explore the biological impact of this regulation, we engineered *S. cerevisiae* and *C. albicans* strains expressing *cdc14*^*S429A*^ and *cdc14*^*S429E*^, or *cdc14*^*S411A*^ and *cdc14*^*S411E*^, respectively, as the sole source of Cdc14 from their natural chromosomal loci. We monitored the recently reported cell wall stress sensitivity phenotypes observed in response to SLiCE motif point mutations (28). Surprisingly, in *S. cerevisiae* and *C. albicans* both the Ala and Glu substitution strains exhibited hypersensitivity to echinocandin drugs that inhibit cell wall synthesis, similar to our previously described *cdc14*^*hm*^ strains harboring Ala substitutions in the SLiCE motif (Fig. 7, *D* and *E*). Comparison of the expression levels of the mutant proteins in *C. albicans* by immunoblotting revealed that the CaCdc14^S411A^ protein was consistently lower than WT CaCdc14, CaCdc14^S411E^, and CaCdc14^hm^, although this difference was not statistically significant (Fig. 7*F*). Thus, in addition to regulating SLiCE motif function, the phosphorylation status of this Ser-Pro Cdk site could influence Cdc14 stability, although this would require additional experimentation to verify. Interestingly, multiple independent isolates of the *C. albicans cdc14*^*S411A*^ and *cdc14*^*S411E*^ strains gave rise to sporadic faster growing colonies that were resistant to the micafungin treatment (Fig. 7*E*). The resistant phenotype was stable after propagation without micafungin, but did not result from reversion, as the Cdk1 site mutations were retained. However, immunoblotting of these resistant strains revealed a consistent and significant increase in Cdc14 expression level (Fig. S4), relative to the original micafungin-sensitive isolates. This is consistent with our prior observations that the cell wall stress phenotype is highly sensitive to modest changes in Cdc14 activity (28) and suggests that cells have complex mechanisms for modulating Cdc14 level and function. Nonetheless, the cell wall stress hypersensitivity caused by the Ser to Glu point mutation is consistent with our biochemical results and those reported previously (41, 42), suggesting that the SLiCE motif has evolved to be under control of Cdk, providing one simple mechanism for cells to dynamically tune Cdc14 activity.

## Discussion

Like the PTP superfamily from which they evolved, Cdc14 enzymes employ a two-step catalytic mechanism (34, 50, 51). The first step involves nucleophilic attack on the substrate phosphoryl group by the thiolate anion of an invariant cysteine within the signature HCX_5_R phosphate binding loop, generating a covalent phosphothioester intermediate (52). This step is facilitated by protonation of the leaving group oxygen by an invariant aspartate, functioning as a general acid. The second step, which is rate-limiting for Cdc14 enzymes (34), involves hydrolysis of the phosphoenzyme intermediate by nucleophilic water activated by the same aspartate, now functioning as a general base. In classical PTPs, a conserved active site glutamine residue is critically important for orienting this hydrolytic water (44, 45, 46). Interestingly, in Cdc14 crystal structures the active site lacks a glutamine in this position, and this may be generally true for DSPs (46). Instead, a nonpolar amino acid at this position forms part of the hydrophobic binding pocket for the substrate +1 Pro that is an essential component of Cdc14 specificity. The mechanism by which these enzymes coordinate water for efficient activation and hydrolysis is unclear.

Here, we have described a novel strategy that the Cdc14 phosphatases evolved to provide an analogous glutamine to the active site to facilitate efficient phosphoenzyme hydrolysis. Cdc14 enzymes contain a pseudosubstrate motif in the disordered C-terminal region, distal to the conserved catalytic domain, which can bind the active site based on AlphaFold2 predictions of numerous Cdc14 orthologs and the kinetic studies presented here. Binding of the pseudosubstrate positions an invariant glutamine in the catalytic pocket, resulting in strong stimulation of the phosphoenzyme hydrolysis rate. Even conservative substitution of asparagine for this glutamine eliminated the stimulatory effect of the fungal SLiCE motif, arguing for specific structural and chemical contributions to the rate-limiting catalytic step, consistent with orienting the attacking water, as in classical PTPs. In support of our model, mutations to the active site Gln446 residue that coordinates the nucleophilic water in the *Yersenia* PTP also resulted in similar decreases in *k*_*cat*_ and *K*_*M*_ like we observed with Cdc14 SLiCE motif mutations (46). This represents an unusual use of a pseudosubstrate motif as most pseudosubstrates inhibit enzymes by blocking substrate access to the catalytic site (53).

Evolution of the SLiCE motif may have been necessitated by the ancient active site reconfiguration that gave Cdc14 enzymes their unique substrate specificity. A signature feature of the Cdc14 family is the tandem DSP domain architecture, where residues from both domains contribute to a substrate binding groove adjacent to the catalytic pocket (37, 38). The novel interface of the DSP domains created binding sites for key Cdc14 substrate features like the +1 Pro and +3 Lys/Arg and an “acidic groove” that favors additional basic amino acids around the +3 position (35, 36). We speculate that this DSP domain duplication, creating the novel active site structure, could have also compromised the catalytic rate for phosphoenzyme hydrolysis, thus selecting for the novel SLiCE motif–mediated stimulation. While the SLiCE motifs are highly conserved in fungi and vertebrate Cdc14 orthologs, it remains unclear if they are a universal feature of all Cdc14 enzymes. We suspect they are, as we also found evidence for SLiCE sequences related to the vertebrate motif in the active sites of AlphaFold2 structures of Cdc14 orthologs from *Drosophila melanogaster* and *Tetrahymena thermophila*. It is interesting that the fungal and vertebrate SLiCE motifs appear unrelated to each other, and it is formally possible that they represent a case of convergent evolution.

An additional putative evolutionary benefit of the SLiCE motif is that its location in the long, disordered C-terminal tail region provided a convenient opportunity for regulation of Cdc14 activity. Fungal Cdc14 orthologs have a highly conserved Ser/Thr-Pro residue adjacent to the SLiCE motif (Fig. 1*A*). This site is known to be phosphorylated in *S. cerevisiae* (41)*, S. pombe* (42), and *C. albicans* (54). In both *S. cerevisiae* and *S. pombe,* evidence suggests that phosphorylation at this site suppresses Cdc14 activity (41, 42). Here, we extended these observations by providing evidence that this site exerts its inhibitory effect by inactivating the SLiCE motif, possibly by disrupting its association with the active site. S429E substitution, mimicking the effect of phosphorylation, reduced *k*_*cat*_ and *K*_*M*_ similar to SLiCE motif mutations, whereas S429A substitution did not. Moreover, treatment of ScCdc14, but not ScCdc14^S429A^, with affinity-purified Cdk1 also reduced *k*_*cat*_ and *K*_*M*_ similarly, consistent with disruption of SLiCE motif function.

In *S. cerevisiae*, where Cdc14 regulation has been most extensively studied, elaborate mechanisms exist to limit Cdc14 activity to a narrow window of the cell division cycle (55). Given its specialized biochemical function in reversing Cdk phosphorylation it is not surprising that Cdc14 activity must be tightly controlled. Many regulatory phosphorylation sites exist in disordered protein loops (56, 57) and evolutionary appearance of the SLiCE motif in the highly disordered C terminus to enhance Cdc14 activity may have enabled the coevolution of phosphoregulation by the kinase that it counteracts. The highly conserved Cdk site adjacent to the fungal SLiCE motif would help keep Cdc14 activity suppressed at times when Cdk activity is high but allow elevation of Cdc14 activity through dephosphorylation of this site at times when Cdk activity is low (42), for example, during anaphase, cytokinesis, and septation when Cdc14 performs many of its known functions. In some species where this inhibitory site is a Ser, its dephosphorylation could be autocatalytic. Cdc14 is activated in certain stress responses in model yeasts (58, 59, 60) and it would be interesting to explore if this site is dephosphorylated under these conditions as part of the activation mechanism. A similar Cdk site is not found adjacent to vertebrate SLiCE motifs and it is unknown if the vertebrate SLiCE motif is also a target for enzyme regulation.

A number of key mechanistic questions remain regarding the SLiCE motif contribution to catalysis. First, if the SLiCE motif binds the enzyme active site, why does it have minimal impact on substrate binding? We propose that the SLiCE motif has much higher affinity for the phosphoenzyme intermediate than for the native enzyme, thus preventing counterproductive inhibition of substrate binding. Consistent with this, several attempts by different methods to directly measure binding affinity of SLiCE peptides to Cdc14 enzymes have failed, even though AlphaFold2 consistently positions this motif in the active site region similar to substrates. Despite the inability to detect a physical interaction in binding assays, these peptides clearly stimulate catalysis and, at least with the fungal SLiCE, can compete with high affinity substrates during catalysis at a step after initial substrate binding. We did find evidence from catalytic efficiency measurements at very low substrate concentrations that the SLiCE motif reduces binding of low affinity substrates. This raises the possibility that one biological function of the SLiCE motif could be to repress nonspecific Cdc14 activity on unintended, low affinity phosphorylation sites, which are likely in great excess over its high affinity sites. This could be a mechanism to further “sharpen” the specificity of Cdc14 *in vivo.* This hypothesis remains to be tested but potentially could be approached with quantitative phosphoproteomics comparing sites affected by WT and SLiCE mutant enzymes.

What would be the basis for selective binding to the phosphoenzyme? The existing crystal structures of hCdc14 and ScCdc14 catalytic domains do not reveal conformational changes between native and substrate-bound forms (37, 38), but we lack structural information on the phosphoenzyme intermediate. A simple explanation is that the active site configuration changes when the phosphate is covalently linked to the catalytic cysteine in a way that significantly increases binding affinity for the SLiCE motif. Another possibility is that the covalently linked phosphate itself contributes binding energy to the SLiCE motif, presumably *via* the invariant SLiCE glutamine, which in the fungal SLiCE motif is the only significant difference compared to a substrate.

The vertebrate slice motif, while using substrate-like features for binding, appears structurally distinct from a substrate, and its predicted alpha helix could simply have stronger affinity for the phosphoenzyme if this state is distinct from the native enzyme. It is noteworthy that we were unable to detect activity of Cdc14 enzymes toward a human SLiCE phosphopeptide containing a phosphoserine in place of the glutamine. This is consistent with the possibility that there is some structural difference in the active site region of the phosphoenzyme that promotes binding of the vertebrate SLiCE alpha helix. However, it is formally possible that addition of the phosphate disrupts the alpha helical structure of this motif or that the human SLiCE phosphopeptide binds the native enzyme similar to the phosphoenzyme, but the phosphate is not oriented appropriately for catalysis, perhaps due to the more rigid nature of the alpha helix compared to the normal disordered structure of optimal substrates. However, we were unable to detect any inhibition of hCdc14A or ScCdc14 activity toward DiFMUP by the human SLiCE phosphopeptide (not shown), arguing against this possibility and suggesting that the vertebrate SLiCE motif has low affinity for the native enzyme relative to the phosphoenzyme. Collectively, our observations are best explained by preferential interaction of the SLiCE motif with the phosphoenzyme intermediate.

While our initial hypothesis was that the SLiCE motif was unique to fungal Cdc14s, the discovery of the vertebrate SLiCE motif and comparison to its fungal counterpart fortuitously led us to evidence of structural and/or mechanistic differences in the active site of fungal and human Cdc14s. Whereas converting the fungal SLiCE sequence into a phosphopeptide resulted in an efficient substrate for both ScCdc14 and hCdc14A, the natural fungal SLiCE sequence only stimulated catalysis by ScCdc14. Similarly, the human SLiCE peptide only efficiently stimulated catalysis of hCdc14A. These data argue for some structural differences between ScCdc14 and hCdc14A active sites, specifically at the phosphoenzyme step of the catalytic cycle. We recently proposed that Cdc14 may be an attractive target for development of antifungal drugs and/or pesticides (29). Cdc14 is broadly required for host infection by fungal pathogens (22, 23, 24, 25, 28). Its structure and substrate specificity appear highly conserved across fungal species and its substrate specificity is somewhat unique among phosphatases (29). While angiosperm plants lack Cdc14 orthologs, Cdc14 is also highly conserved in metazoans. In mammals, current evidence based on KO mice suggest that it is not required for normal postnatal growth and development (21), and other reported phenotypes associated with loss of Cdc14 function support that side effects associated with temporary inhibition to prevent or treat an invasive fungal infection might be minimal (14, 16, 19, 61). Nonetheless, it would be ideal to develop inhibitor compounds that are selective for fungal Cdc14s and our results provide the first evidence that this may be feasible.

## Experimental procedures

### Strain and plasmid construction

All plasmids and strains used in this study are listed in Tables S2 and S3, respectively. All engineered *S. cerevisiae* and *C. albicans* strains were confirmed by PCR and DNA sequencing. *S. cerevisiae* strains YKA1039 and YKA1040 were created using the *delitto perfetto* approach exactly as described (62). *C. albicans* strains HCAL133 and HCAL134 were created by integration of a single copy of *cdc14*^*S411A*^*-3xHA* or *cdc14*^*S411E*^*-3xHA* by transformation of a SalI/EcoRV fragment of pHLP738 or pHLP739, respectively, into one *cdc14* locus of JC8 (*cdc14Δ/Δ*) and selecting on synthetic medium lacking uracil. *Escherichia coli* expression plasmids for purification of recombinant Cdc14 enzymes were generated using the Gateway cloning system (Invitrogen). Codon-optimized coding sequences for *Homo sapiens CDC14A* (codons 1-413) and *CDC14B* (codons 1-411) were synthesized by Twist Bioscience. All expression sequences were initially inserted into pENTR/D-TOPO and then transferred to either pDEST17 (ScCdc14 expression) or pDEST15 (hCdc14A and hCdc14B expression) destination vectors for expression with N-terminal 6xHis or glutathione-*S*-transferase (GST) tag, respectively. Mutations were generated by QuikChange (Agilent) or In-Fusion (Takara Bio) systems. Full coding sequences for all plasmids were verified by WideSeq.

### Cell culture

*S. cerevisiae* and *C. albicans* cultures were grown in yeast extract/peptone/dextrose (YPD) medium (10 g/l yeast extract, 20 g/l peptone, and 20 g/l glucose) at 30 °C with shaking at 225 rpm. *C. albicans* strains were supplemented with 8 μg/ml uridine. *E. coli* were grown in 2xYT (16 g/l tryptone, 10 g/l yeast extract, and 5 g/l NaCl) at 37 °C with shaking at 225 rpm. Agar was added to 20% (w/v) for growth on solid medium. For agar spotting assays, liquid cultures were grown to saturation, serially diluted in 8-fold steps starting from *A*_600_ = 1.0, and 5 μl of four consecutive dilutions were spotted on YPD plates ± 20 ng/ml micafungin and grown at 30 °C for 3 to 5 days. For *C. albicans* patch assays, single colonies were resuspended in 100 μl PBS and spread on YPD agar plates ± 50 ng/ml micafungin.

### Protein expression and purification

6xHis-ScCdc14 enzymes were purified on 1 ml HisTrap nickel sepharose columns (Cytiva) as previously described (29). GST-tagged hCdc14A and hCdc14B enzymes were purified in batch on Glutathione Superflow agarose resin (Thermo Fisher Scientific) as described (34). Peak fractions were pooled and dialyzed overnight into 25 mM Hepes pH 7.5, 300 mM NaCl, 2 mM EDTA, 0.1% 2-mercaptoethanol, and 40% glycerol prior to storage at −80 °C in small aliquots. Protein purity was assessed by SDS-PAGE with Coomassie blue staining and protein concentration was determined by Bradford assay for 6xHis-ScCdc14 enzymes or from SDS-PAGE using a serum albumin standard curve and ImageLab software (Bio-Rad; https://www.bio-rad.com/en-us/product/image-lab-software?ID=KRE6P5E8Z) for GST-hCdc14A and hCdc14B enzymes (due to contamination with free GST in hCdc14B preparations).

### Structural prediction and modeling

Protein structures were predicted with the AlphaFold2 algorithm (43) *via* the Google Colaboratory interface (63), using default settings. Protein structure visualization, superposition, and modeling were performed using Molecular Operating Environment software (Chemical Computing Group; https://www.chemcomp.com/en/Products.htm).

### Steady-state enzyme kinetics

Activity of Cdc14 enzymes under steady-state conditions toward varying concentrations of DiFMUP (Invitrogen) was assayed continuously on a Biotek Cytation 1 plate reader in Cdc14 reaction buffer (25 mM Hepes pH 7.5, 150 mM NaCl, 2 mM EDTA, and 2 mM tris(2-carboxyethyl)phosphine) at 30 °C as previously described (29). Data from velocity *versus* substrate concentration plots were fit with the Michaelis–Menten equation in GraphPad Prism (https://www.graphpad.com/) to determine steady-state kinetic parameters *k*_*cat*_ and *K*_*M*_. For *trans* peptide stimulation assays, DiFMUP was used at a single concentration well above the measured *K*_*M*_ for each enzyme. Synthetic peptides containing WT and mutant variants of the fungal and vertebrate SLiCE motifs were synthesized by Genscript and purified in-house by C18 reversed-phase chromatography. Activity under steady-state conditions towards varying concentrations of the Acm1pS3 phosphopeptide, MI(pS)PSKKRTIL (where pS is phosphoSer), were assayed in Cdc14 reaction buffer with Biomol Green (Enzo Life Science) detection of released inorganic phosphate as previously described (29). Data were fit with a modified Michaelis–Menten equation including a substrate inhibition term in Prism. Acm1pS3 and phosphopeptide variants of the fungal and human SLiCE motifs were synthesized by Genscript and purified in-house.

### Presteady-state enzyme kinetics

Presteady-state kinetic analysis was performed as described previously (34). Enzymes were exchanged into Cdc14 reaction buffer using a desalting column and flash-frozen in liquid nitrogen. Prior to use, aliquots were thawed and clarified at 16,000*g* for 5 min to remove any insoluble material and protein concentration was subsequently determined by Bradford assay. Presteady-state measurements were made by mixing enzyme and varying DiFMUP concentrations at 23 °C on an SFM-3000 stopped-flow instrument equipped with a MOS-200/M spectrophotometer (BioLogic) as described (64). The excitation wavelength was 390-nm and emission was selected using a 409-nm long pass filter (Semrock FF02-409/LP-25). A standard curve was generated by measuring the fluorescence of defined concentrations of the product DiFMU reconstituted in Cdc14 reaction buffer under identical conditions. Raw data were fit with Equation 1 in Prism:

(1) $$Y=\text{At}+B\left(1-e^{-\text{bt}}\right)+C$$

This provided rates for the burst phase (b) or *k*_*burst*_. Plots of *k*_*burst*_ *versus* DiFMUP concentration were fit with Equation 2:

(2) $$k_{\text{burst}}=\left(k_{2}+k_{3}\right)\left[S\right]/\left(K_{D}+\left[S\right]\right)$$

### Microscale thermophoresis

A Monolith NT Label Free instrument (NanoTemper) was used to measure binding of a synthetic peptide containing a noncleavable (α,α-difluoromethylene)phosphonoserine residue (pCF_2_-Ser) within an optimal Cdc14 substrate sequence (EV(pCF_2_-Ser)PTKR), which we synthesized previously (29), to Cdc14 enzymes. Label-free binding assays were performed in triplicate at room temperature in 50 mM Tris–HCl pH 8.0, 150 mM NaCl, 5% glycerol, 0.5 mM DTT, 0.1% pluronic F127 with 20% excitation power and medium MST power. Data were analyzed using the MO.Affinity Analysis software (NanoTemper; https://shop.nanotempertech.com/en/software/analysis-software). Background was subtracted from the fluorescence signal change at different synthetic peptide concentrations and the values converted to fraction bound by dividing by curve amplitude. Fraction bound was plotted as a function of ligand concentration and fit with a standard dose-response function in GraphPad Prism to determine the dissociation constant, *K*_*D*_.

### Catalytic efficiency measurements

Cdc14 catalytic efficiencies toward a pooled set of phosphopeptide variants were measured by LC-MS exactly as described (49). Briefly, phosphopeptides with unique masses were synthesized and purified (GenScript) and subsequently pooled (final concentration of 375 nM each phosphopeptide) in Cdc14 reaction buffer. Reactions were initiated by addition of Cdc14 enzymes (0.1–1 μM final concentration) in 200 μl Cdc14 reaction buffer and incubated at 30 °C for 10 to 40 min 60 μl aliquots were removed and mixed with an equal volume 5% formic acid at desired times to terminate the reactions. Peptides were desalted on C18 spin columns (Thermo Fisher Scientific) and dried by vacuum centrifugation prior to LC-MS analysis on an LTQ Orbitrap Velos mass spectrometer. Extracted ion chromatograms for each substrate and product peptide were generated and integrated using Skyline software (65) (https://skyline.ms/project/home/begin.view) and catalytic efficiency calculations from the data were performed as described (28, 47).

### Clb2-Cdk immunoprecipitation

Cdk1 (Clb2-Cdc28) enzyme was immunoaffinity isolated on goat anti-rabbit IgG magnetic beads (Thermo Fisher Scientific) from a *S. cerevisiae sic1Δ* strain harboring a high copy plasmid with galactose-inducible *CLB2* fused to the ZZ domain from protein A exactly as described previously (66). A mock purification from cells lacking the expression plasmid served as a negative control. Activity of bead-bound Cdk in kinase buffer (100 mM Hepes (pH 7.5), 50 mM NaCl, 10 mM MgCl_2_, 0.5 mM DTT, and 1 mM ATP) was initially validated on 100 μM synthetic peptide NELRSPSKRRS (30 °C for 12h) by MALDI-TOF mass spectrometry. Then, 5 μM purified recombinant Cdc14 enzymes were incubated with Cdk or mock beads in kinase buffer under identical reaction conditions. Beads were removed by centrifugation and the treated Cdc14 enzymes were used in steady-state kinetic assays with DiFMUP as described above.

### Immunoblotting

Immunoblotting with mouse anti-hemagluttinin (1:5000; Sigma-Aldrich, 12CA5) and rabbit anti-PSTAIR (1:5000; Millipore-Sigma, 06-923) primary antibodies and horseradish peroxidase-conjugated goat anti-mouse and anti-rabbit secondary antibodies (1:10,000, Jackson ImmunoResearch, 115-035-003 and 111-035-003) was conducted exactly as described (28). Quantification was performed using ImageLab software (Bio-Rad) with signals first normalized to the anti-PSTAIR load control.

## Data availability

Raw and processed data files are publicly available *via* the Purdue University Research Repository at DOI: 10.4231/429K-QE94. The corresponding author may also be contacted with data access inquiries.

## Supporting information

This article contains supporting information (26, 28).

## Conflicts of interest

The authors declare that they have no conflicts of interest with the contents of this article.
