# Supplementary material for: Cdc14 phosphatases use an intramolecular pseudosubstrate motif to stimulate and regulate catalysis
Source: J Biol Chem. 2024 Aug 8;300(9):107644. doi: 10.1016/j.jbc.2024.107644 (PMC11407943; doi:10.1016/j.jbc.2024.107644)
Supplement: Supporting Figures and Tables [file mmc1.pdf]

Table S1: Kinetic parameters of wild-type and SLiCE mutant Cdc14 enzymes towards DiFMUP at pH 7.5 and 30 °C

**Enzyme**	k_{cat} **(sec^{-1})**	K_M **(µM)**
ScCdc14$^{1\text{-}449}$	1.96 ± 0.24	8.4 ± 1.6
ScCdc14$^{1\text{-}374}$	0.126 ± 0.003	0.76 ± 0.13
ScCdc14$^{P433A/K435A}$	0.27 ± 0.03	1.0 ± 0.3
ScCdc14^{Q432A}	0.037 ± 0.002	0.72 ± 0.34
ScCdc14^{Q432E}	0.031 ± 0.003	1.11 ± 0.22
ScCdc14^{Q432N}	0.029 ± 0.004	0.92 ± 0.36
hCdc14A$^{1\text{-}413}$	2.63 ± 0.35	24 ± 3
hCdc14A^{Q395A}	0.35 ± 0.03	2.6 ± 0.3
hCdc14A^{3A}	0.065 ± 0.02	0.52 ± 0.30
hCdc14B$^{1\text{-}411}$	4.70 ± 0.18	46 ± 15
hCdc14B^{Q390A}	0.30 ± 0.05	2.88 ± 0.32
hCdc14B^{3A}	0.146 ± 0.005	1.68 ± 0.61
ScCdc14$^{1\text{-}449}$ (Fig. 6)	1.71 ± 0.24	25 ± 3
ScCdc14^{S429A}	1.89 ± 0.22	34 ± 6
ScCdc14^{S429E}	0.15 ± 0.01	3.3 ± 0.2

Table S2: Plasmids used

Plasmid name	Backbone	Marker	Expressed protein	Source
pHLP695	pDEST17		6xHis-ScCdc14$^{1\text{-}449}$	Ref 28
pHLP690	pDEST17		6xHis-ScCdc14$^{1\text{-}374}$	Ref 28
pHLP621	pDEST17		6xHis-ScCdc14$^{P433A/K435A}$	Ref 28
pHLP622	pDEST17		6xHis-ScCdc14^{Q432A}	Ref 28
pHLP623	pDEST17		6xHis-ScCdc14^{Q432E}	This study
pHLP766	pDEST17		6xHis-ScCdc14^{Q432N}	This study
pHLP674	pDEST15		GST-hCdc14A$^{1\text{-}413}$	This study
pHLP730	pDEST15		GST-hCdc14A^{Q395A}	This study
pHLP731	pDEST15		GST-hCdc14A^{3A}	This study
pHLP678	pDEST15		GST-hCdc14B$^{1\text{-}411}$	This study
pHLP732	pDEST15		GST-hCdc14B^{Q390A}	This study
pHLP733	pDEST15		GST-hCdc14B^{3A}	This study
pHLP764	pDEST17		6xHis-ScCdc14^{S429A}	This study
pHLP765	pDEST17		6xHis-ScCdc14^{S429E}	This study
pHLP738	pFA-URA3	*URA3*	CaCdc14^{S411A}-3xHA	This study
pHLP739	pFA-URA3	*URA3*	CaCdc14^{S411E}-3xHA	This study

All plasmids have ampicillin resistance marker for *E. coli*.

Table S3: Yeast strains used

Strain name	Species	*Relevant genotype*	Source
YKA1038	*S. cerevisiae*	*cdc14*^{P433A,K435A}	Ref 28
YKA1039	*S. cerevisiae*	*cdc14*^{S429A}	This study
YKA1040	*S. cerevisiae*	*cdc14*^{S429E}	This study
JC8	*C. albicans*	*cdc14::hisG/cdc14::hisG*	Ref 26
JC2721	*C. albicans*	*CDC14-3xHA*:*URA3*/*cdc14::hisG*	Ref 28
HCAL102	*C. albicans*	*cdc14*$^{Q414A/P415A/K417A}$*-3xHA*:*URA3*/*cdc14::hisG*	Ref 28
HCAL133	*C. albicans*	*cdc14*^{S411A}*-3xHA*:*URA3*/*cdc14::hisG*	This study
HCAL134	*C. albicans*	*cdc14*^{S411E}*-3xHA*:*URA3*/*cdc14::hisG*	This study

All *S. cerevisiae* strains are derived from the W303 genetic background (MATα *ade2-1 can1-100 his3-11,15 leu2-3,112 trp1-1 ura3-1*)

All *C. albicans* strains are derived from the CAI4 genetic background (*ura3::imm434/ura3::imm434*)

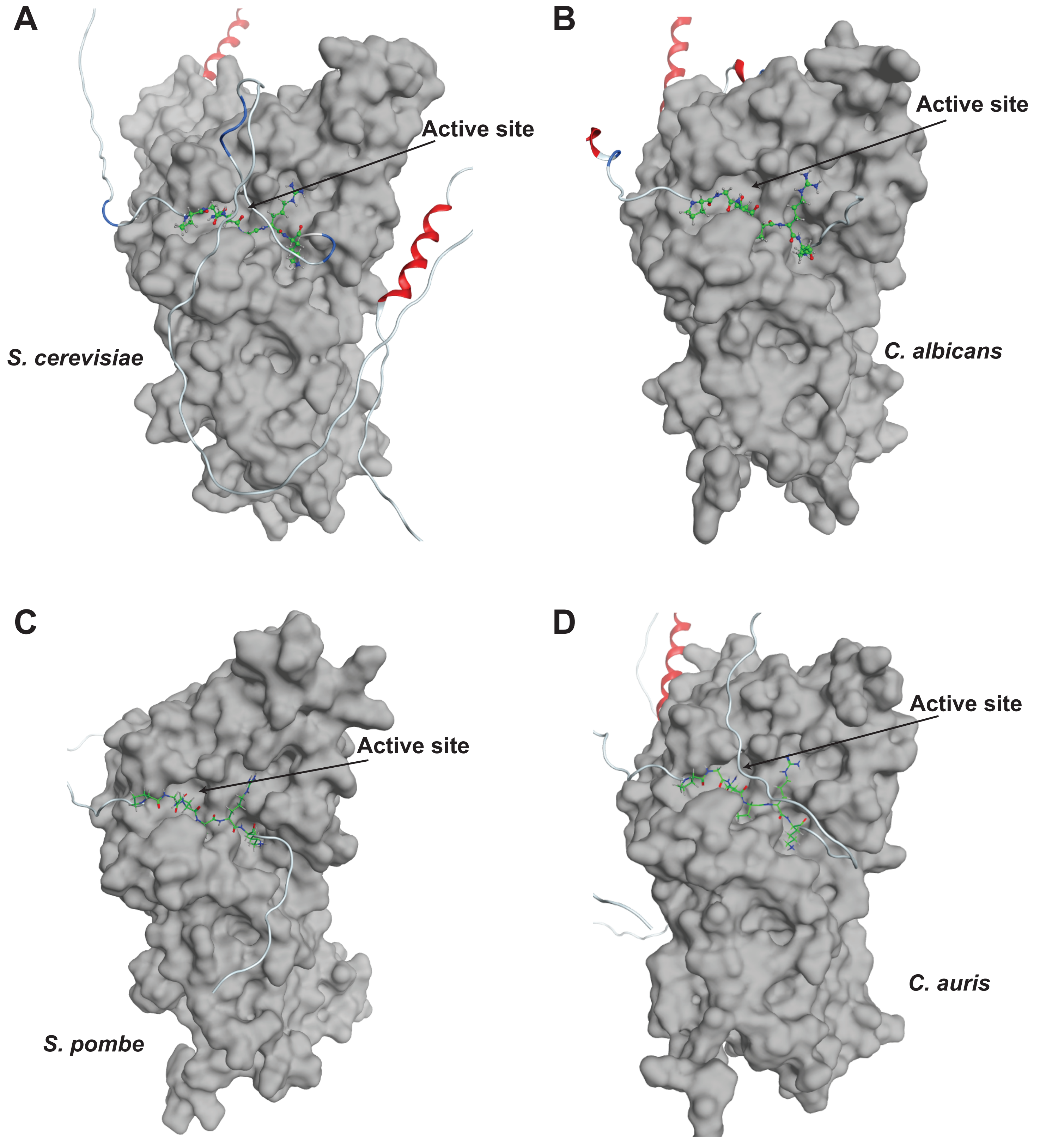

Figure S1. AlphaFold2 structure predictions of Cdc14 orthologs from representative fungal species. Roughly equivalent poses relative to the active site are shown. The conserved catalytic domain is rendered in surface mode whereas the non-conserved C-terminal region is rendered with secondary structure ribbon mode. Stick mode with green backbone is used for the region containing the conserved SLiCE motif, found docked in the active site in each case. The sequence of Candida albicans Cdc14 was truncated after the SLiCE motif prior to modeling.

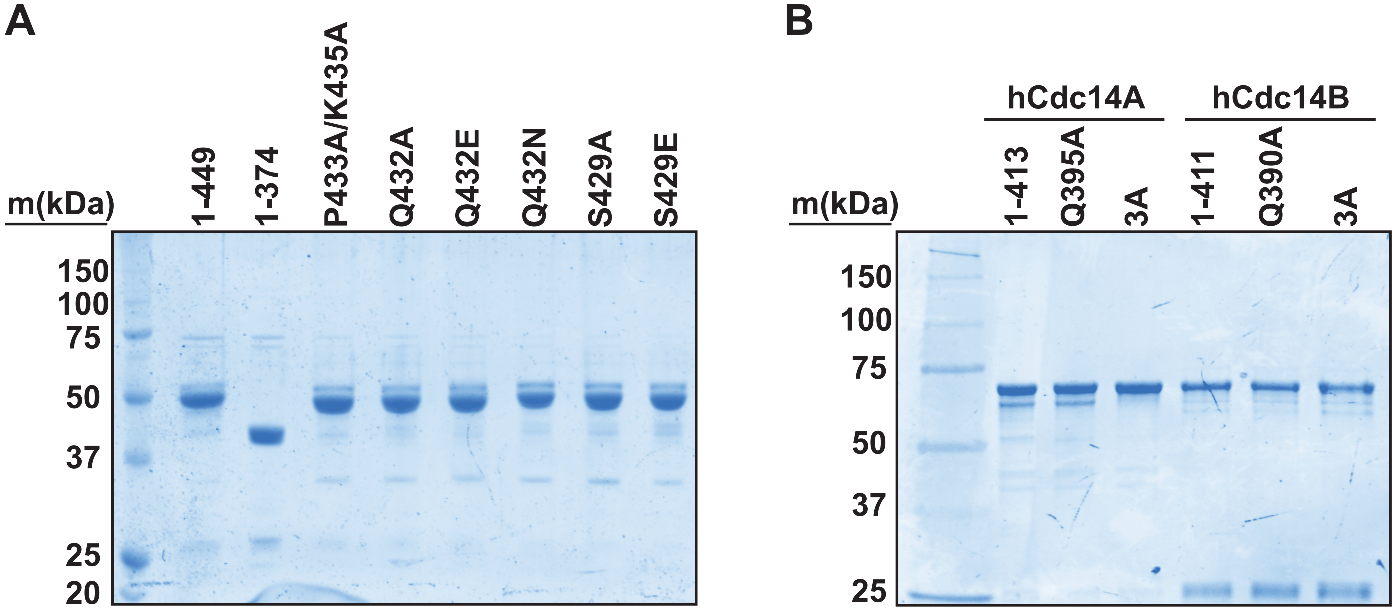

Figure S2. Recombinant Cdc14 protein purifications. **(A)** All 6His-ScCdc14 variants used in the study were compared by 10% SDS-PAGE stained with Coomassie blue. 2 µg each protein were loaded. (**B)** All GST-hCdc14A and B variants used in the study were similarly compared, loading 2 µg each protein.

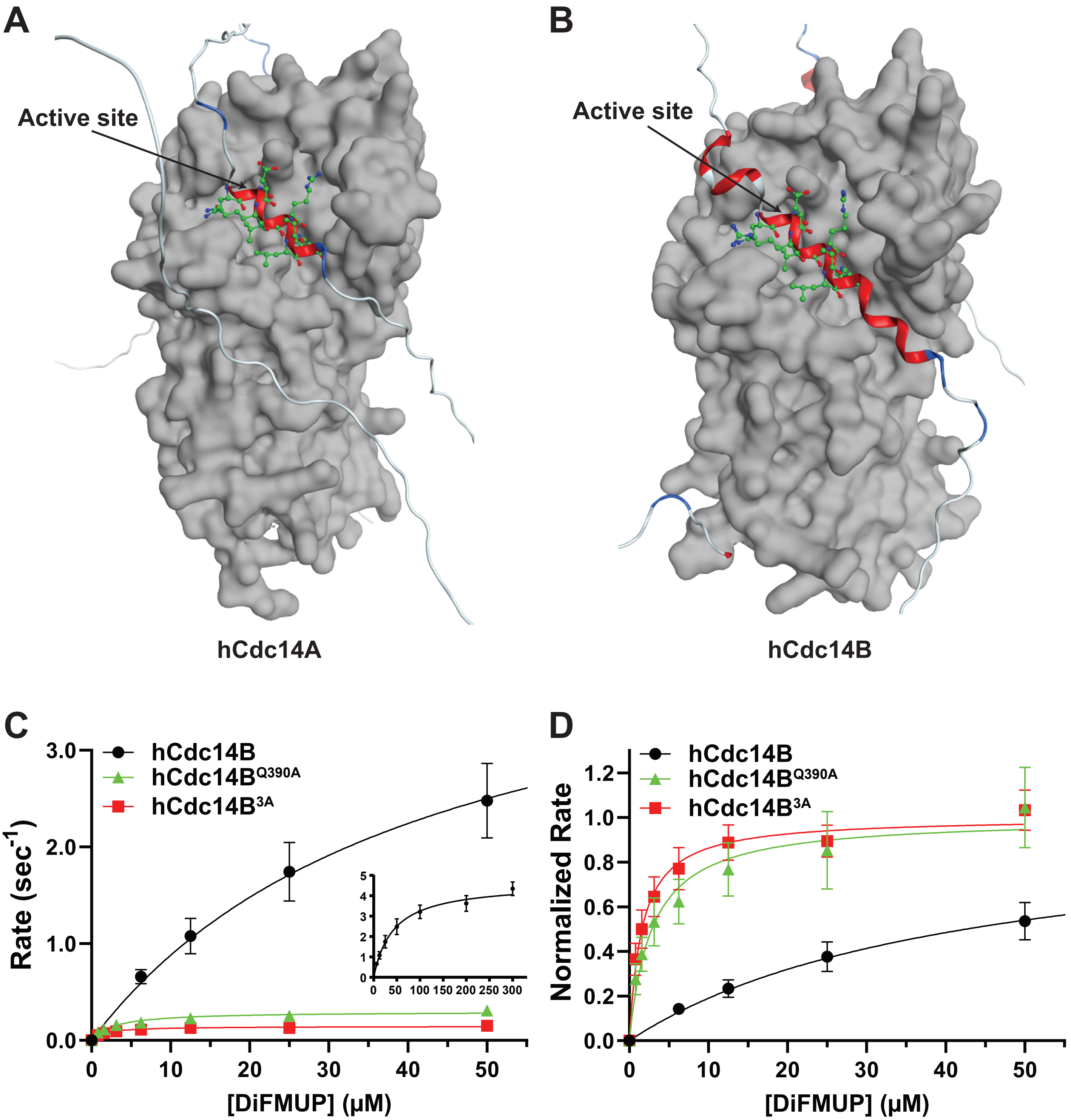

Figure S3. AlphaFold2 structure predictions of hCdc14A and hCdc14B and kinetic analysis of hCdc14B SLiCE variants. **(A-B)** Same as Figure S1 for human Cdc14 orthologs. **(C-D)** Steady state kinetic analysis of hCdc14B and its SLiCE motif mutant derivatives exactly as described in Figure 5D-E for hCdc14A.

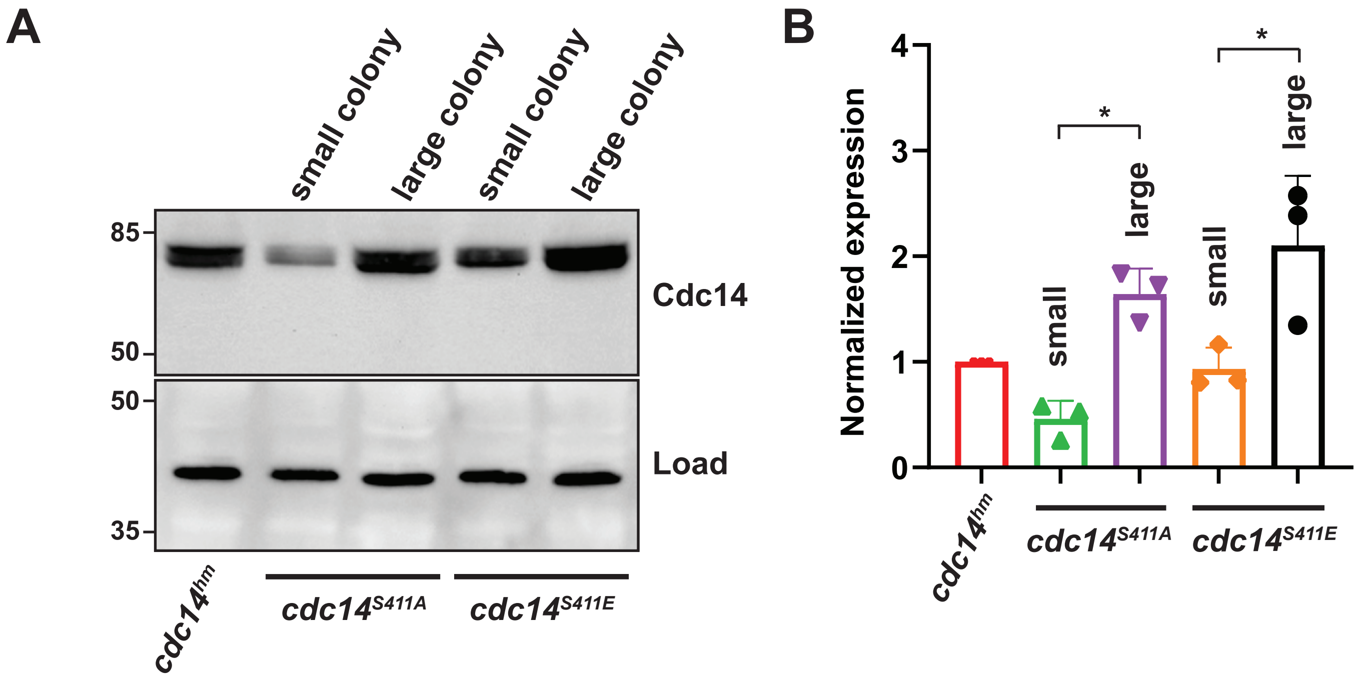

Figure S4. Resistant derivatives of *C. albicans cdc14S411A* and *cdc14S411E* strains have elevated Cdc14 expression. **(A)** Immunoblotting of Cdc14 level in cell extracts from original (small) and fast-growing (large) isolates of the indicated strains compared to the previously described *cdc14*^{hm} strain (28). Samples were prepared from identical log phase YPD liquid cultures and blots probed with anti-HA antibody (to detect Cdc14) and anti-PSTAIR (loading control). Numbers at left are size markers, in kDa. The blot is representative of three experiments with independent isolates that are quantified in panel B. **(B)** Quantitation of immunoblotting of three independent fast-growing (large) derivatives of *cdc14S411A* and *cdc14S411E* compared to three independent original (small) isolates and to *cdc14*^{hm}. Bars are means with standard deviations and individual data points are shown. Expression levels are normalized to those of *cdc14*^{hm}. * = p-value < 0.05 in one way ANOVA with Tukey post hoc test comparing the large and small strain variants.
